# Distinct signatures of dental plaque metabolic byproducts dictated by periodontal inflammatory status

**DOI:** 10.1038/srep42818

**Published:** 2017-02-21

**Authors:** Akito Sakanaka, Masae Kuboniwa, Ei Hashino, Takeshi Bamba, Eiichiro Fukusaki, Atsuo Amano

**Affiliations:** 1 Department of Preventive Dentistry, Osaka University Graduate School of Dentistry, 1–8 Yamadaoka, Suita, Osaka 565–0871, Japan; 2 AMED-CREST, Japan Agency for Medical Research and Development, 1-7-1 Otemachi, Chiyoda-ku, Tokyo, 100-0004, Japan; 3 Project ‘Challenge to Intractable Oral Disease’, Osaka University Dental Hospital; 1-8 Yamadaoka, Suita, Osaka 565-0871, Japan; 4 Division of Metabolomics, Research Center for Transomics Medicine, Medical Institute of Bioregulation, Kyushu University, 3-1-1 Maidashi, Higashi-ku, Fukuoka 812-8582, Japan; 5 Department of Biotechnology, Osaka University Graduate School of Engineering, 2–1 Yamadaoka, Suita, Osaka 565–0871, Japan

## Abstract

Onset of chronic periodontitis is associated with an aberrant polymicrobial community, termed dysbiosis. Findings regarding its etiology obtained using high-throughput sequencing technique suggested that dysbiosis holds a conserved metabolic signature as an emergent property. The purpose of this study was to identify robust biomarkers for periodontal inflammation severity. Furthermore, we investigated disease-associated metabolic signatures of periodontal microbiota using a salivary metabolomics approach. Whole saliva samples were obtained from adult subjects before and after removal of supragingival plaque (debridement). Periodontal inflamed surface area (PISA) was employed as an indicator of periodontal inflammatory status. Based on multivariate analyses using pre-debridement salivary metabolomics data, we found that metabolites associated with higher PISA included cadaverine and hydrocinnamate, while uric acid and ethanolamine were associated with lower PISA. Next, we focused on dental plaque metabolic byproducts by selecting salivary metabolites significantly decreased following debridement. Metabolite set enrichment analysis revealed that polyamine metabolism, arginine and proline metabolism, butyric acid metabolism, and lysine degradation were distinctive metabolic signatures of dental plaque in the high PISA group, which may be related to the metabolic signatures of disease-associated communities. Collectively, our findings identified potential biomarkers of periodontal inflammatory status and also provide insight into metabolic signatures of dysbiotic communities.

Chronic marginal periodontitis, the sixth most prevalent infectious disease worldwide^1^, causes inflammatory destruction to alveolar bone, with tooth loss the final stage. The worldwide financial burden of severe periodontitis has been estimated to be $53.99B^2^. Despite its high prevalence and adverse effects on quality of life, periodontitis is usually diagnosed in a later stage, when patients develop observable periodontal inflammation and destruction of alveolar bone. Thus, there is urgent need for an easy method to detect disease activity in earlier stages to allow for intervention prior to disease progression. Along this line, analysis of the saliva metabolome, which represents the collection of metabolites in saliva, has been proposed as an effective tool for periodontal diagnosis, since collection of saliva is easy, safe, and cost effective, with several studies reporting salivary metabolic profiles related to periodontitis using categorical classification of the disease^3^,^4^. We previously presented a prediction model of periodontal inflammation severity that uses a combination of salivary metabolomics and *periodontal inflamed surface area* (PISA), a quantitative description of the burden of periodontal inflammation^5^, which highlighted the utility of PISA for salivary metabolomics research^6^. Additionally, by removing supragingival plaque and calculus (debridement) to minimize background metabolites, we identified potential salivary metabolites that reflected the severity of periodontal inflammation, mostly in patients with moderate periodontitis. The first aim of the present study, for which we recruited a larger number of patients as compared to our former investigation, specifically those with severe periodontitis, was to perform a more robust exploration of biomarkers of periodontal inflammation that can be used for rapid periodontitis screening without debridement as a part of a routine health check.

The other important finding from our previous study is that debridement results in decreased levels of particular metabolites in saliva. Dental plaque is known to possess a distinct fluidic component, termed dental plaque fluid, that can be separated by centrifugation of a plaque sample^7^. It has also been shown that plaque fluid contains various metabolites as byproducts of microbial metabolism in the community^8^. This observation suggested that metabolic byproducts are eluted from dental plaque into saliva, which seems to reflect the microbial metabolism within dental plaque. Therefore, elucidation of salivary metabolites that diminish after debridement will likely help to identify metabolic byproducts in dental plaque and provide insight into its microbial metabolism.

In addition, next generation sequencing techniques have been used to describe the composition of the periodontal polymicrobial community in great depth and previous studies have revealed the presence of an imbalance in relative abundance of members of the community associated with periodontitis, termed dysbiosis, in affected patients^9^,^10^. Current models of periodontitis progression hold that periodontal inflammation and dysbiosis positively reinforce each other regardless of which was the original cause^11^. This notion indicates that the severity of periodontal inflammation can reveal the presence of the dysbiotic community. Furthermore, the dysbiotic microbiota in periodontitis patients was shown to exhibit a highly conserved metatranscriptome signature in several metabolic pathways, despite high inter-patient variability in terms of taxonomic composition^12^, suggesting that the conserved metabolic signature is an emergent property of the dysbiotic microbiome in periodontitis progression.

We speculated that metabolic signatures associated with the dysbiotic community can be explained by periodontal inflammation severity and, in particular, distinct signatures of metabolic byproducts from dental plaque can be observed in patients with high PISA values. Thus, the second aim of this study was to examine metabolic byproducts from dental plaque present in saliva by comparing the salivary metabolome between before and after debridement, and also investigate how these metabolites are impacted by differences in PISA.

## Results

### Demographic and clinical characteristics of the subjects

A total of 50 subjects, with a median age of 45.0 years [interquartile range (IQR) 38.0–51.8), were enrolled. Their clinical parameters are listed in Table 1. As expected, PISA (median 360.0, IQR 202.6–608.3) had significantly positive correlations with periodontal epithelial surface area^5^ (PESA; median 1197.5, IQR 1056.6–1370.4; r = 0.823; *p* < 0.01), and the definition of periodontitis provided by the Centers for Diseases Control and Prevention (CDC) and American Academy of Periodontology (AAP)^13^ (r = 0.604, *p* < 0.01). Modified plaque index (mPlI; median 4.2; IQR 2.4 to 5.4; for calculation see Methods) also showed correlations with PISA (r = 0.615, *p* < 0.01), PESA (r = 0.461, *p* < 0.01), and CDC-AAP case definitions (r = 0.373, *p* < 0.01). Meanwhile, CDC-AAP case definitions had a significantly positive correlation with age (r = 0.316, *p* = 0.025).

### Strong correlation between PISA and salivary metabolome

We performed metabolic profiling of 100 saliva samples obtained during both pre- and post-debridement collection from the 50 subjects using gas chromatography/mass spectrometry (GC/MS), and identified 69 metabolites, including 5 trimethylsilyl (TMS)-derivatives of identical metabolites; alanine, fucose, isoleucine, leucine, and serine. The metabolites identified are listed in Supplementary Table S1.

Before analyzing the relationship between PISA and salivary metabolites, we evaluated the strength of correlation between each clinical parameter and the pre-debridement salivary metabolic profile. For this, we conducted a series of orthogonal projection to latent structures (OPLS) regression analyses, where metabolome information was employed as an explanatory variant to evaluate each clinical parameter as a response variant. Eventually, OPLS model quality was assessed through interpretation of model parameters [i.e., number of components, *R*^2^, *Q*^2^, cross-validated ANOVA (CV-ANOVA) *p* value]. Of the clinical parameters tested, PISA was shown to be the most reliable for explaining the variation of salivary metabolic profile, suggesting that the saliva metabolome is strongly influenced by periodontal inflammatory status (Table 2).

### Metabolites associated with periodontal inflammatory status

**Figure 1:**
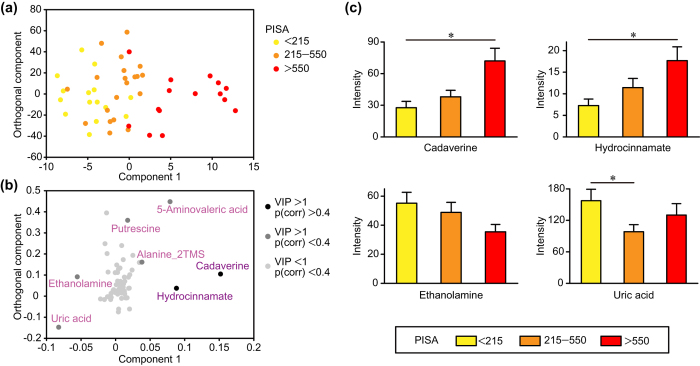
Salivary metabolites associated with periodontal inflammatory status. (**a**) Score plot of OPLS model for PISA. Each point represents a single pre-debridement saliva sample from each of the 50 subjects, color-coded based on PISA value. Component 1 denotes the variance of the salivary metabolic profile related to PISA. Variance unrelated to PISA was filtered into the orthogonal component. (**b**) Loading plot of OPLS model for PISA. Each point represents a single metabolite and its position corresponds to the PISA value, with those on the right associated with higher PISA. Shading of circles corresponds to significance in construction of the model, with darker circles indicating a stronger contribution. (**c**) Differential metabolite abundance in saliva based on PISA value. Data are presented as the mean ± SE and a Kruskal-Wallis rank sum test was used for statistical analysis. *p < 0.05.

**Figure 2:**
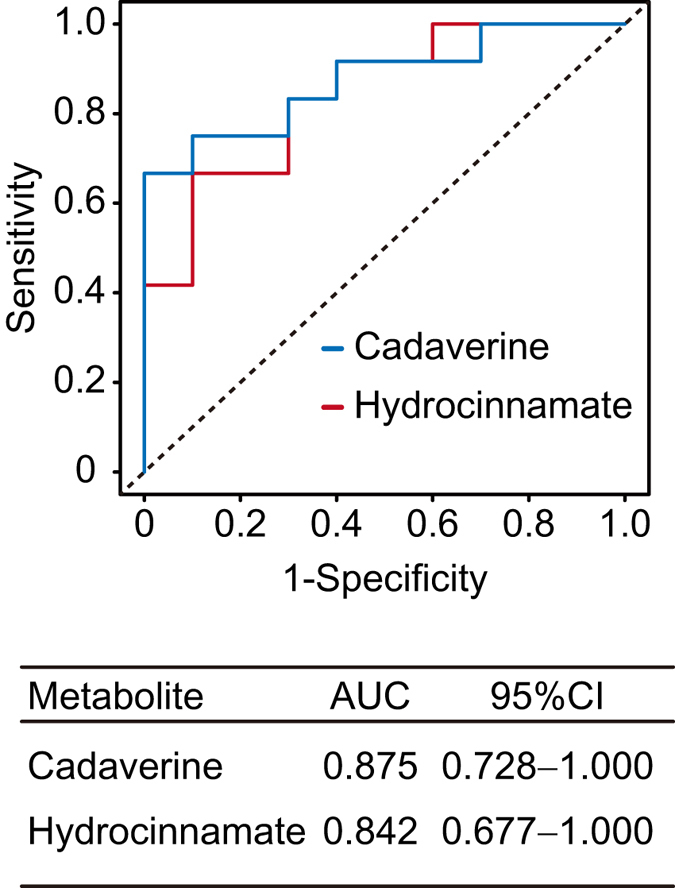
Receiver operating characteristic (ROC) curves for cadaverine and hydrocinnamate used to identify severe periodontitis. The area under the curve (AUC), as well as ROC curves for cadaverine and hydrocinnamate used to identify severe periodontitis are presented.

Next, we analyzed the OPLS model for PISA in order to elucidate relationships between PISA and salivary metabolites. In the score plot shown in Fig. 1a, each point represents a single sample and color-coding is based on PISA score, with the right side indicating higher PISA. The loading plot showed metabolite distribution corresponding to PISA (Fig. 1b). In order to determine significant metabolites associated with high or low PISA, we employed a combination of variable importance in projection (VIP) > 1 and p(corr) >0.4 values, which were regarded as the equivalent of *p* < 0.05 in univariate analysis^14^. As a result, cadaverine and hydrocinnamate were found to be linked to higher PISA with high statistical significance. We also noted that uric acid and ethanolamine were related to lower PISA with moderate statistical significance, while 5-aminovaleric acid, alanine_2TMS, and putrescine were related to higher PISA. Indeed, univariate analyses confirmed that salivary levels of cadaverine and hydrocinnamate were increased in subjects with higher PISA, while the opposite trends were observed for ethanolamine and uric acid (Fig. 1c). Furthermore, receiver operating characteristic (ROC) curves indicated that cadaverine and hydrocinnamate yielded satisfactory accuracy for diagnosis of severe periodontitis [area under the curve (AUC) = 0.875 and 0.842, respectively] (Fig. 2).

### Exploration of metabolic byproducts from dental plaque

**Figure 3:**
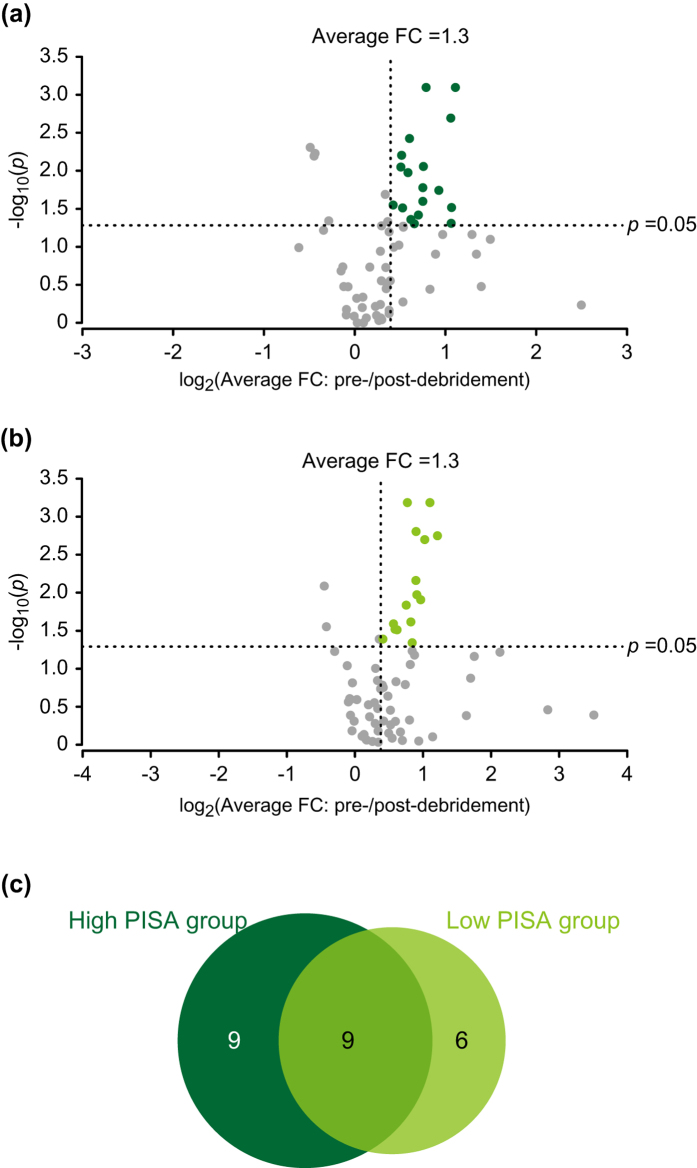
Debridement-induced shift in salivary metabolite abundance in high and low PISA groups. (**a**) Volcano plot of relative changes of 69 salivary metabolites in high PISA group. Metabolite abundance shift is plotted on the x-axis as the log_2_ average fold difference between pre- and post-debridement saliva samples, while −log_10_ (*p* values) are plotted on the y-axis. Significantly reduced metabolites via debridement were selected with cutoff values of average fold change >1.3 and *p* value < 0.05. (**b**) Volcano plot of relative changes of 69 salivary metabolites in low PISA group. (**c**) Venn diagram of significantly reduced metabolites in high and low PISA groups. Metabolite numbers are depicted for each category. FC: fold change.

Our OPLS analysis demonstrated that pre-debridement saliva contained several metabolites, which reflected the status of periodontal inflammation. To investigate the extent to which supragingival microbial metabolism contributes to production of these metabolites, we compared their salivary levels between before and after debridement with the aim of exploring metabolic byproducts in supragingival plaque. We then compared the differences of those metabolic byproducts between the high and low PISA groups (n = 15 each). Metabolites significantly reduced by debridement were selected using average fold change >1.3 and *p* < 0.05 as the criteria. As a result, we selected 18 metabolites in the high and 15 in the low PISA groups (Fig. 3, Tables 3, 4). Of those, 9 metabolites were specific to the high PISA group, which included cadaverine, hydrocinnamate, 5-aminovaleric acid, and putrescine, while 6 were specific to the low PISA group, including ethanolamine. These results suggested that all metabolites selected by OPLS analysis, except for uric acid, had an association with microbial metabolism.

### Metabolic signatures of dental plaque in high PISA group

When we focused on metabolites specific to the high PISA group, amino acid catabolites and polyamines appeared to be dominant. To further characterize the signatures of the selected metabolites in that group, we conducted metabolite set enrichment analysis (MSEA), which is able to identify which pathways are overrepresented among selected metabolites. Based on 64 identified metabolites (without consideration of derivatization difference), high-PISA-specific metabolites were subjected to statistical hypothesis testing of cross-tabulation using Fisher’s exact test^15^ in reference to a metabolite set list created on the basis of *The Kyoto Encyclopedia of Genes and Genomes* (Supplementary Table S2). As shown in Table 5, polyamine metabolism, arginine and proline metabolism, butyric acid metabolism, and lysine degradation were identified as significantly overrepresented pathways in the high PISA group (*p* = 0.000925, 0.017558, 0.017857, and 0.049539, respectively). These findings suggest that the metabolic activities of these pathways are upregulated in supragingival microbiota of individuals with high PISA.

## Discussion

In this study, we constructed a comprehensive prediction model to demonstrate the relationship between PISA and pre-debridement salivary metabolic profiles, which not only validated our previous findings, but presented novel biomarkers including those associated with periodontal health. Furthermore, we investigated debridement-induced changes in the salivary metabolome associated with periodontal inflammation severity in an attempt to explore surrogate indicators for metabolic signatures of dysbiotic communities. The most notable difference between the current study and our earlier pilot trial is that the focus of that previous investigation centered on detecting metabolites derived from subgingival area, while the present focused more on detecting microbial-derived metabolites, from which metabolic signatures of the dysbiotic community were inferred.

As the first step of the present study, we constructed a prediction model of PISA using pre-debridement salivary metabolic profile as an explanatory variant. Our results identified cadaverine and hydrocinnamate as highly specific salivary markers for periodontal inflammation severity (Fig. 1). The greater abundance of cadaverine in subjects with higher PISA is intriguing, as other previous metabolomics analyses also demonstrated a significant increase in levels of cadaverine in saliva^4^ and gingival crevicular fluid (GCF)^16^ of periodontitis patients. Cadaverine production has been found to be associated with putrefaction and attributed exclusively to bacterial decarboxylation of lysine^17^. Furthermore, a human gingivitis study reported that dental biofilm accumulation during oral hygiene restriction enhanced the production of cadaverine, leading to an increase in its salivary level^18^. Cadaverine was also identified as a potential indicator of periodontal inflammation severity in our previous pilot study. It is of particular importance to note that the association of PISA with salivary level of cadaverine was detected without debridement in the present study, which had a greater number of subjects with severe periodontitis, while our previous study, whose subjects mostly consisted of moderate cases, required analysis of post-debridement saliva samples in order to narrow down candidate metabolites that reflect periodontal inflammation severity. This might be attributed to characteristics seen in severe periodontitis such as deeper periodontal pockets, from which a large amount of cadaverine is released, or changes in the metabolic activities of supragingival microbiota. In either case, we consider that cadaverine is likely a robust biomarker for periodontal inflammation severity regardless of debridement and could be used for rapid periodontitis screening. On the other hand, the relationship of hydrocinnamate, an analogue of phenylalanine, to periodontal pathogenesis remains largely unknown. A previous study showed that hydrocinnamate was produced from polyphenols by gut microbiota^19^, suggesting a microbial origin, while another noted that periodontitis patients exhibited increased salivary levels of aromatic amino acid metabolites^20^. Therefore, phenylalanine metabolism is likely to be upregulated in the disease-associated community, leading to an elevated salivary level of hydrocinnamate in individuals with high PISA.

Other metabolites associated with higher PISA in our OPLS analysis included 5-aminovaleric acid, putrescine, and alanine_2TMS (Fig. 1b), though the association of alanine_2TMS with higher PISA is unclear, since differences in derivatization efficiency could influence the results. Interestingly, the other metabolites are known to be associated with cadaverine. For example, it has been reported that 5-aminovaleric acid is involved in bacterial catabolism of cadaverine^21^,^22^, while this metabolite is considered to be a bacterial waste product and its higher salivary level in periodontal disease has also been documented^23^. Putrescine, categorized as a polyamine along with cadaverine, is produced from decarboxylation of ornithine and an increased level of putrescine in the GCF of periodontitis patients has been reported^16^. It is noteworthy that both cadaverine and putrescine are produced from amino-acid decarboxylation reactions, which result in consumption of cytoplasmic protons and generation of a proton motive force, leading to energy generation^24^. Considering that several metabolites of amino-acid decarboxylation, including cadaverine and putrescine, have been shown to be associated with bacterial vaginosis, a major dysbiosis-related disease^25^, the abundance of amino acid decarboxylases in the microbial community seems to indicate enhanced bacterial activities of the dysbiosis-related community.

Conversely, uric acid and ethanolamine were identified as differential metabolites in the lower PISA group (Fig. 1b). Uric acid is a well-known salivary antioxidant^26^ and its salivary level has been shown to be decreased in periodontitis patients^16^,^27^, which was supported by our findings. It has also been reported that neutrophils from periodontitis patients exhibit hyperactivity in terms of generation of reactive oxygen species^28^, suggesting that host responses involve elevated oxidative status in periodontitis patients, thus leading to a lower salivary level of uric acid. Meanwhile, until recently, little was known about the roles of ethanolamine in periodontal pathogenesis. However, a metatranscriptome study that used small noncoding RNAs revealed that ethanolamine catabolism is a tightly-regulated pathway during periodontitis progression, suggesting its role in transition of the microbial community from a commensal to dysbiotic state^29^. In another study, ethanolamine in the gastrointestinal tract was shown to enhance the growth of enteric pathogens, while the resident microbiota did not efficiently metabolize ethanolamine^30^. Periodontal pathogens may also exploit ethanolamine as a noncompetitive metabolite to outgrow oral microbes associated with periodontal health, leading to a significant decrease in its salivary volume in individuals with high PISA.

As the second step of this work, we explored metabolic byproducts from supragingival plaque obtained from the high and low PISA groups, then characterized possible metabolic signatures of supragingival plaque in the high group; that is, polyamine metabolism, arginine and proline metabolism, butyric acid metabolism, and lysine degradation (Table 5).

Although we removed only supragingival plaque and left subgingival plaque intact, with the intention of avoiding bleeding during debridement, our results seem to partially represent the metabolic signatures of the dysbiotic community as a whole. During the process of dental plaque maturation, microbes embedded deep in matured supragingival plaque likely grow under anaerobic conditions and are involved in the microbial shift to dysbiosis. Indeed, previously presented evidence shows a phylogenetic similarity or co-occurrence pattern of specific periodontal pathogens between supragingival and subgingival plaque^31^,^32^,^33^. Furthermore, the vast majority of putative virulence factors up-regulated in periodontitis were reported to be expressed by organisms in oral biofilm considered to be non-pathogenic, suggesting that the community as a whole becomes more virulent during the process of disease development^34^. Thus, it seems plausible that our results from MSEA reflect an emergent property of dominant metabolic pathways in dysbiotic communities as a whole. Indeed, a recent microbiome study showed enrichment of the metagenome of genes involved in amino acid catabolism, including arginine, proline, and lysine, as well as polyamine synthesis (spermidine) in subgingival plaque from periodontitis patients^35^. Amino acid catabolism has also been implicated to be associated with dysbiotic communities based on metatranscriptome analysis of subgingival plaque^36^. Specifically, lysine degradation has been frequently found to be associated with disease-associated communities in conjunction with butyric acid metabolism in both metagenome^37^ and metatranscriptome^12^ analyses of subgingival plaque. Given that transition from periodontal health to disease is linked to perturbations in overall functional output, including altered metabolic signatures in a periodontal microbial community, these findings raise the possibility that salivary metabolites are surrogate indicators for metabolic signatures of the community, which would be helpful for monitoring disease activity as well as untangling the complex microbial interactions that occur during formation of a periodontopathic community.

Meanwhile, we acknowledge that gender distribution in the present study was not optimal, as there were more males in the high PISA group and more females in the low PISA group. As a result, we have provided data regarding the impact of the imbalance in gender distribution on the results as supplementary information. The score plots of principal component analysis showed a poor separation between male and female subjects within the respective high and low PISA groups (Supplementary Figs S1 and S2), indicating that gender had little effect on variations in salivary metabolic profiles in both groups. Furthermore, statistical comparative analyses demonstrated that there were few gender-specific differences in pre-debridement salivary metabolite levels or their average fold changes induced by debridement (Supplementary Tables S3 and S4). Accordingly, we consider that the impact of the imbalance in gender distribution on the results was negligibly small as compared to that induced by the differences in PISA.

In summary, results of this study identified differential salivary metabolites related to periodontal inflammatory status and suggest possible metabolic signatures of supragingival plaque associated with periodontal inflammation severity. These findings may be useful to develop new diagnostic markers of periodontitis, while they will also lead to a better understanding of conserved metabolic signatures of dysbiotic communities in individuals affected by periodontitis.

## Methods

### Study design

Fifty volunteers aged from 31 to 69 years (25 males, 25 females) were enrolled in this study, which was conducted at Osaka University Hospital from November 2013 to March 2014. All participants provided written consent for participation and publication, and the same exclusion criteria were used as in the pilot study^6^. The Ethical Committee for Clinical Research of Osaka University Graduate School of Dentistry approved all study protocols, which were conducted in accordance with the principles of the Declaration of Helsinki. This study conformed to the STROBE guidelines for human observational studies.

### Periodontal examination and saliva collection

Participants were asked to refrain from brushing and using mouthwash for at least 1 h prior to undergoing a periodontal examination and saliva collection, which were performed as in the pilot study. Based on a definition of plaque index^38^, plaque accumulation was measured at four sites per tooth for all teeth present and the average per tooth of the total scores was defined as mPlI. Periodontal inflammation severity was evaluated using PISA, while the definition of periodontitis provided by the CDC and AAP^13^ was used, the same as in the pilot study.

Unstimulated whole saliva samples were collected between 13:00 and 15:00, then again at 15-minute intervals after debridement performed with an ultrasonic scaler. To distinguish the metabolites of microbial origins from those related to the host, we avoided insertion of the scaler tip into the gingival sulcus in an attempt to avoid bleeding. Finally, 1 mL of each sample was immediately frozen with liquid nitrogen and stored at −80 °C until analysis.

### Salivary metabolomics analysis

We used a previously reported method for sample preparation and GC/MS analysis^6^. Briefly, we employed a GCMS-QP2010 Ultra (Shimadzu, Kyoto, Japan) equipped with an InertCap 5MS/NP capillary column (30 m × 0.25 mm i.d. with 0.25-μm film thickness, GL Science Inc., Tokyo, Japan) and an AOC-20i autosampler (Shimadzu). Peaks were detected and aligned using MetAlign software^39^. Obtained data were processed by AIoutput software in order to produce a peak matrix^40^, then the peaks were annotated by comparing retention indices and unique mass spectra, with the reference library prepared from authentic standards. The mass spectra of other peaks not compared with standards were compared with those of the NIST11 MS library, which was also used to confirm the results of annotations provided by AIoutput. The assigned peak intensities were normalized against the peak intensity of ribitol, the internal standard.

### Statistical analysis

A series of OPLS regression analyses were performed to construct prediction models of clinical parameters [PISA, PESA, case definition for periodontitis, mPlI] using the salivary metabolic profile as an explanatory variant. Briefly, PISA represents the bleeding (inflamed) surface area of pocket epithelium, while PESA is the surface area of both healthy and inflamed pocket epithelia^5^. All models were constructed using pareto scaling. In order to compare the strength of the associations between the salivary metabolic profile and clinical parameters, we employed parameters indicative of model quality (number of components, *R*^2^, *Q*^2^, CV-ANOVA *p* value)^14^. Briefly, *R*^2^ represents the goodness of fit, while *Q*^2^ indicates the predictive ability of the model. The number of components is related to the degree of overfitting. The CV-ANOVA *p* value can be used as a measure of significance for the observed model construction. Metabolites that contributed most to constructing a prediction model of PISA were chosen based on a combination of VIP value >1 and a p(corr) value >0.4^14^. Values for the AUC of the ROC curve were used to assess the diagnostic ability of selected metabolites for diagnosis of severe periodontitis. Comparative analyses of debridement-induced shift in salivary metabolite abundance in the high and low PISA groups (n = 15 each) were conducted using a paired *t* test or Wilcoxon signed rank test depending on normality distribution. For MSEA, we selected metabolites that were decreased by debridement using values obtained from the combination of average fold change >1.3 and *p* value < 0.05. Using these metabolites, MSEA was performed as previously described^15^. Spearman correlation analysis was used to assess correlations among the obtained clinical parameters. All univariate statistical analyses were performed with SPSS (version 22; IBM Japan), while all multivariate statistical analyses were performed using SIMCA-P software (version 13.0; Umetrics, Umeå, Sweden).

## Additional Information

**How to cite this article**: Sakanaka, A. *et al*. Distinct signatures of dental plaque metabolic byproducts dictated by periodontal inflammatory status. *Sci. Rep.* **7**, 42818; doi: 10.1038/srep42818 (2017).

**Publisher's note:** Springer Nature remains neutral with regard to jurisdictional claims in published maps and institutional affiliations.

## Supplementary Material

Supplementary Tables and Figures

## Figures and Tables

**Table 1:** Demographic and clinical characteristics of the subjects.

	CDC-AAP case definitions			Total
	No Periodontitis	Moderate periodontitis	Severe periodontitis	
Number of participants	10 (20%)	28 (56%)	12 (24%)	50 (100%)
Age, years (range)	37.5 (34.0–42.5)	45.0 (38.0–53.8)	49.0 (44.8–52.0)	45.0 (38.0–51.8)
Sex, female	8 (80%)	14 (50%)	3 (25%)	25 (50%)
PISA (range)	199.3 (155.5–252.8)	302.5 (200.5–449.4)	930.2 (537.7–1238.5)	360 (202.6–608.3)
PESA (range)	1048 (994.3–1124.0)	1196.4 (1099.3–1265.6)	1660.5 (1462.7–1762.3)	1197.5 (1056.6–1370.4)
mPlI (range)	2.4 (1.7–4.1)	4.2 (3.0–5.4)	5.3 (4.1–7.3)	4.2 (2.4–5.4)

Data are shown as number (%) or median (IQR).

**Table 2:** Quality parameters for each OPLS model.

Variable	*R*^2^	*Q*^2^	Number of components	CV-ANOVA *p* value
PISA	0.798	0.502	5	0.000937
PESA	0.755	0.376	5	0.0279
Case definitions	0.676	0.242	5	0.295
mPlI	0.456	−0.0277	3	1
Sample ID	0.156	−0.0588	1	1

**Table 3:** Metabolites significantly reduced by debridement in high PISA group.

Metabolites	Average FC in peak intensity (pre-/post-debridement)	*p* value
**4-Aminobutyric acid**	1.73	8.05E-04
5-Oxoproline	2.16	8.05E-04
**Cadaverine**	2.08	2.04E-03
Hypotaurine	1.52	3.77E-03
**Phenylalanine**	1.43	6.29E-03
Aspartic acid	1.69	8.82E-03
**5-Aminovaleric acid**	1.42	8.99E-03
**Succinic acid**	1.50	1.06E-02
Indole-3-acetic acid	1.68	1.67E-02
Glutamic acid	1.90	1.81E-02
Alanine_3TMS	1.68	2.54E-02
**Putrescine**	1.34	2.83E-02
Leucine	2.10	3.06E-02
N-Acetylornithine	1.44	3.09E-02
**Hydrocinnamate**	1.63	3.83E-02
**Ornithine**	1.54	4.39E-02
Fucose_2	2.09	4.93E-02
**Fructose 6-phosphate**	1.58	4.98E-02

High-PISA-specific metabolites are shown in boldface type. FC: fold change.

**Table 4:** Metabolites significantly reduced by debridement in low PISA group.

Metabolites	Average FC in peak intensity (pre-/post-debridement)	*p* value
5-Oxoproline	2.15	6.55E-04
Aspartic acid	1.71	6.55E-04
**Tryptophan**	1.87	1.57E-03
**Glutamine**	2.32	1.79E-03
Fucose_2	2.04	2.01E-03
Glutamic acid	1.86	6.94E-03
Indole-3-acetic acid	1.88	1.07E-02
N-Acetylornithine	1.96	1.25E-02
**Isoleucine_1TMS**	1.69	1.46E-02
**Fucose_1**	1.77	2.43E-02
**Ethanolamine**	1.48	2.57E-02
Leucine	1.52	3.04E-02
Alanine_3TMS	1.53	3.08E-02
Hypotaurine	1.33	4.09E-02
**Alanine_2TMS**	1.79	4.57E-02

Low-PISA-specific metabolites are shown in boldface type. FC: fold change.

**Table 5:** Characterization of high PISA specific pathways based on MSEA.

Metabolic pathway	*p* value
Polyamine metabolism	9.25E-04
Arginine and proline metabolism	1.76E-02
Butyric acid metabolism	1.79E-02
Lysine degradation	4.95E-02
Glutathione metabolism	5.17E-02
Phenylalanine metabolism	9.16E-02
Tyrosine metabolism	1.41E-01
Glutamate, glutamine metabolism	1.41E-01
Citrate cycle (TCA cycle)	3.70E-01
Fructose and mannose metabolism	3.70E-01
Glycolysis/gluconeogenesis	3.70E-01
Pentose phosphate pathway	4.63E-01
Phenylalanine, tyrosine, tryptophan biosynthesis	4.63E-01
