# Supplementary material for: Distinct signatures of dental plaque metabolic byproducts dictated by periodontal inflammatory status
Source: Sci Rep. 2017 Feb 21;7:42818. doi: 10.1038/srep42818 (PMC5318866; doi:10.1038/srep42818)
Supplement: Supplementary Tables and Figures [file srep42818-s1.pdf]

Supplementary Information for:

Distinct signatures of dental plaque metabolic byproducts dictated by periodontal inflammatory status

Akito Sakanaka[1], Masae Kuboniwa[1, 2, *], Ei Hashino[1, 3], Takeshi Bamba[4, 5], Eiichiro Fukusaki[2, 5], Atsuo Amano[1]

[1]Department of Preventive Dentistry, Osaka University Graduate School of Dentistry, 1–8 Yamadaoka, Suita, Osaka 565–0871, Japan

[2]AMED-CREST, Japan Agency for Medical Research and Development, 1-7-1 Otemachi, Chiyoda-ku, Tokyo, 100-0004, Japan

[3]Project 'Challenge to Intractable Oral Disease', Osaka University Dental Hospital; 1-8 Yamadaoka, Suita, Osaka 565-0871, Japan

[4]Division of Metabolomics, Research Center for Transomics Medicine, Medical Institute of Bioregulation, Kyushu University, 3-1-1 Maidashi, Higashi-ku, Fukuoka 812-8582, Japan

[5]Department of Biotechnology, Osaka University Graduate School of Engineering, 1–8 Yamadaoka, Suita, Osaka 565–0871, Japan

***Corresponding author**:

Email: kuboniwa@dent.osaka-u.ac.jp

Supplementary Table S1. Peak information of metabolites in saliva

Peak No.	RT (sec)	RI	Quantitative *m/z*	Compound Name	KEGG compound	Peak area: before debridement Average	before debridement S. D.	after debridement Average	after debridement S. D.	Ratio (after/before)	*p* value[a]	
1	273.3	1001.154	117	Propylene glycol	C00583	2.41	1.73	2.69	1.92	1.1	0.331	
2	305.6	1048.92	174	Oxalacetic acid + Pyruvate	C00022+ C00036	13.12	8.73	13.97	6.29	1.1	0.509	
3	344.2	1106.248	116	Alanine_2TMS	C00041	48.69	33.23	35.81	21.58	0.7	0.005	*
4	360.8	1131.794	131	2-Hydroxybutyrate	C05984	2.35	1.74	3.29	2.33	1.4	0.001	*
5	366.9	1141.182	116	Sarcosine	C00213	4.16	2.66	4.73	3.88	1.1	0.278	
6	377.9	1158.11	86	Leucine_1TMS	C00123	7.59	6.22	4.18	2.13	0.6	0.000	*
7	390.5	1177.501	130	2-Aminobutyric acid	C02261	1.87	1.02	2.00	0.92	1.1	0.327	
8	391.7	1179.347	86	Isoleucine_1TMS	C00407	3.31	3.63	1.46	0.93	0.4	0.001	*
9	419.1	1223.115	144	Valine	C00183	17.23	14.16	16.03	10.84	0.9	0.511	
10	443.3	1263.128	132	Serine_2TMS	C00065	7.14	5.67	6.37	4.75	0.9	0.385	
11	451	1275.86	174	Ethanolamine	C00189	44.47	27.28	40.70	22.23	0.9	0.306	
12	453	1279.167	158	Leucine_2TMS	C00123	12.76	11.49	12.38	7.95	1.0	0.810	
13	466.7	1301.959	158	Isoleucine_2TMS	C00407	5.26	6.03	3.18	2.55	0.6	0.015	*
14	469.5	1306.944	142	Proline	C00148	63.92	60.24	84.27	81.34	1.3	0.060	
15	474.5	1315.848	147	Succinic acid	C00042	70.45	51.43	52.33	28.95	0.7	0.026	*
16	475.4	1317.45	174	Glycine	C00037	186.60	112.46	159.41	98.87	0.9	0.127	
17	487.9	1339.708	189	Glyceric acid	C00258	1.39	1.26	1.15	0.88	0.8	0.128	
18	504.6	1369.444	204	Serine_3TMS	C00065	6.82	6.85	3.70	3.76	0.5	0.002	*
19	506.3	1372.472	188	Alanine_3TMS	C00041	5.59	4.83	3.34	2.12	0.6	0.001	*
20	520.5	1397.756	218	Threonine	C00188	2.14	1.62	1.67	0.85	0.8	0.027	*
21	527	1409.981	255	Thymine	C00178	1.17	0.76	1.10	0.52	0.9	0.466	

22	534.1	1423.505	104	Hydrocinnamate_NIST	C05629	11.67	9.98	11.05	9.03	0.9	0.337	
23	541.2	1437.029	248	b-Alanine	C00099	1.24	0.58	1.03	0.50	0.8	0.010	*
24	589.1	1530.163	232	Aspartic acid	C00049	19.99	15.90	12.24	8.39	0.6	0.000	*
25	590.7	1533.415	156	5-Oxoproline	C01879	47.14	25.60	25.19	10.57	0.5	0.000	*
26	594.8	1541.748	174	4-Aminobutyric acid	C00334	2.47	1.72	1.77	1.36	0.7	0.000	*
27	610.6	1573.862	115	Creatinine	C00791	1.56	1.19	1.62	1.04	1.0	0.611	
28	622.5	1598.049	193	3-Phenyllactic acid	C05607	1.27	0.66	1.08	0.51	0.9	0.013	*
29	631.1	1616.451	188	Hypotaurine	C00519	2.78	1.88	2.27	1.41	0.8	0.005	*
30	636.8	1628.725	246	Glutamic acid	C00302	33.20	29.07	18.04	12.01	0.5	0.000	*
31	642.4	1640.784	174	5-Aminovaleric acid	C00431	424.98	235.97	340.20	176.38	0.8	0.009	*
32	643.1	1642.291	218	Phenylalanine	C00079	18.51	11.89	13.83	6.81	0.7	0.002	*
33	645.4	1647.244	179	4-Hydroxyphenylacetic acid	C00642	6.65	5.84	6.04	5.19	0.9	0.206	
34	648.3	1653.488	257	Lauric acid	C02679	0.79	0.53	1.41	1.08	1.8	0.000	*
35	669.8	1699.785	103	Ribose	C00121	2.25	2.19	2.17	1.94	1.0	0.839	
36	694.4	1755.707	117	Fucose_1	C01018	79.62	76.14	47.21	36.73	0.6	0.002	*
37	695.7	1758.663	174	Putrescine	C00134	154.88	104.38	122.79	77.66	0.8	0.005	*
38	700.4	1769.35	117	Fucose_2	C01018	21.29	22.31	10.83	10.07	0.5	0.000	*
39	707.4	1785.266	156	Glutamine	C00064	16.35	17.86	7.96	5.82	0.5	0.001	*
40	715.5	1803.868	174	O-Phosphoethanolamine	C00346	29.97	27.42	37.56	25.85	1.3	0.011	*
41	721.4	1817.956	204	Shikimic acid	C00493	2.26	1.89	2.15	1.90	0.9	0.743	
42	722.1	1819.628	265	Hypoxanthine	C00262	2.41	1.99	3.06	2.06	1.3	0.094	
43	729.5	1837.297	142	Ornithine_NIST	C00077	47.84	36.23	35.74	24.03	0.7	0.008	*
44	730.1	1838.73	273	Isocitric acid + Citric acid	C00311+ C00158	14.87	15.61	21.12	14.23	1.4	0.000	*
45	737.9	1857.354	174	Cadaverine	C01672	45.57	37.10	35.83	30.10	0.8	0.019	*
46	741.6	1866.189	194	Caffeine	C07481	3.36	3.02	3.10	2.79	0.9	0.253	

47	746	1876.695	217	1,5-Anhydro glucitol	C07326	3.14	2.10	4.36	2.29	1.4	0.000	*
48	749.3	1884.575	264	Adenine	C00147	1.12	0.58	1.13	0.51	1.0	0.903	
49	761.4	1914.157	103	Fructose	C00085	3.34	13.69	0.74	1.64	0.2	0.183	
50	766.3	1926.456	205	Galactose	C00124	15.47	8.80	11.95	7.30	0.8	0.004	*
51	768.8	1932.731	205	Glucose	C00031	14.00	17.67	19.52	20.52	1.4	0.011	*
52	771.4	1939.257	174	Lysine	C00047	40.71	27.10	35.22	23.11	0.9	0.151	
53	771.7	1940.01	154	Histidine	C00135	14.81	11.98	12.21	10.23	0.8	0.204	
54	778.8	1957.831	218	Tyrosine	C00082	82.69	47.31	71.88	36.29	0.9	0.104	
55	788.1	1981.175	237	Paraxanthine	C13747	3.80	3.18	5.11	4.23	1.3	0.002	*
56	789.2	1983.936	202	Indole-3-acetic acid	C00954	3.43	3.12	2.06	1.53	0.6	0.000	*
57	799.4	2009.974	174	N-Acetylornithine	C00437	16.70	20.92	10.81	10.24	0.6	0.026	*
58	810.1	2038.058	353	Xanthine	C00385	1.98	1.94	2.59	2.17	1.3	0.135	
59	841.2	2120.593	205	N-Acetylgalactosamine	C00203	1.08	0.97	1.01	0.89	0.9	0.621	
60	842.9	2125.261	441	Uric acid	C00366	116.50	71.67	130.39	72.67	1.1	0.094	
61	846	2133.773	217	Inositol	C00137	7.99	3.40	8.79	3.12	1.1	0.062	*
62	850.2	2145.305	315	Arabinose-5-phosphate	C01112	1.07	0.78	0.98	0.53	0.9	0.428	
63	887.6	2250.143	202	Tryptophan	C00078	2.58	2.38	1.31	0.64	0.5	0.000	*
64	900.4	2286.862	144	Spermidine	C00315	1.99	1.50	1.86	1.20	0.9	0.449	
65	929	2371.744	387	Fructose 6-phosphate	C00085	1.45	1.19	1.11	0.75	0.8	0.056	
66	1030.3	2695.909	246	N-Acetylneuraminate	C00270	8.83	7.12	8.79	7.82	1.0	0.969	
67	1054.6	2779.972	204	b-Lactose	C01970	0.97	1.09	2.07	3.24	2.1	0.015	*
68	1074.5	2850.609	361	Maltose	C00208	3.89	3.48	4.82	4.15	1.2	0.183	
69	1074.8	2851.685	204	Lactitol	C13542	1.62	3.25	1.19	2.11	0.7	0.091	

[a]* $p < 0.05$

Supplementary Table S2. Metabolite set list

Metabolic pathway tested	Metabolites classified in each metabolic pathway[a]
Alanine, aspartate, asparagine metabolism	Alanine, Aspartic acid
Arginine and proline metabolism	4-Aminobutyric acid, 5-Aminovaleric acid, Creatinine, Glutamic acid, Ornithine, Proline, Putrescine, Sarcosine, Spermidine
Biotin metabolism	Lysine, Pyruvate
Butyric acid metabolism	4-Aminobutyric acid, Succinic acid
Caffeine metabolism	Caffeine, Paraxanthine, Xanthine
Citrate cycle (TCA cycle)	Citric acid, Pyruvate, Succinic acid
Cysteine and methionine metabolism	Alanine, Aspartic acid, Pyruvate, Serine
Fructose and mannose metabolism	Fructose, Fructose 6-phosphate, Fucose
Glutamate, glutamine metabolism	4-Aminobutyric acid, 5-Oxoproline, Glutamic acid, Glutamine, Succinic acid
Glutathione metabolism	5-Oxoproline, Cadaverine, Glutamic acid, Glycine, Ornithine, Putrescine, Spermidine
Glycine, serine and threonine metabolism	Aspartic acid, Glyceric acid, Glycine, Sarcosine, Serine, Threonine, Tryptophan
Glycolysis / Gluconeogenesis	Fructose 6-phosphate, Glucose, Pyruvate
Glyoxylate and dicarboxylate metabolism	Citric acid, Glutamine, Glyceric acid, Glycine, Pyruvate, Serine
Histidine metabolism	Aspartic acid, Histidine
Lysine biosynthesis	Aspartic acid, Lysine
Lysine degradation	5-Aminovaleric acid, Cadaverine, Lysine
Nicotinic acid metabolism	Aspartic acid, Pyruvate
Pentose phosphate pathway	Fructose 6-phosphate, Glucose, Glyceric acid, Pyruvate
Phenylalanine metabolism	3-Phenyllactic acid, 4-Hydroxyphenylacetic acid, Hydrocinnamate, Phenylalanine
Phenylalanine, tyrosine and tryptophan biosynthesis	Phenylalanine, Shikimic acid, Tryptophan, Tyrosine
Polyamine metabolism	4-Aminobutyric acid, Cadaverine, Ornithine, Putrescine, Spermidine
Propionic acid metabolism	2-Hydroxybutyrate, b-Alanine, Propyleneglycol

Purine metabolism	Adenine, Glutamine, Glycine, Hypoxanthine, Xanthine
Pyrimidine metabolism	b-Alanine, Glutamine, Thymine
Pyruvate metabolism	Pyruvate
Tryptophan metabolism	Indole-3-acetic acid, Tryptophan
Tyrosine metabolism	4-Hydroxyphenylacetic acid, Phenylalanine, Pyruvate, Succinic acid, Tyrosine
Valine, leucine and isoleucine biosynthesis	Isoleucine, Leucine, Threonine, Valine
Valine, leucine and isoleucine degradation	Isoleucine, Leucine, Valine

[a]Detected metabolites are listed with high-PISA-specific metabolites gray-highlighted in each pathway.

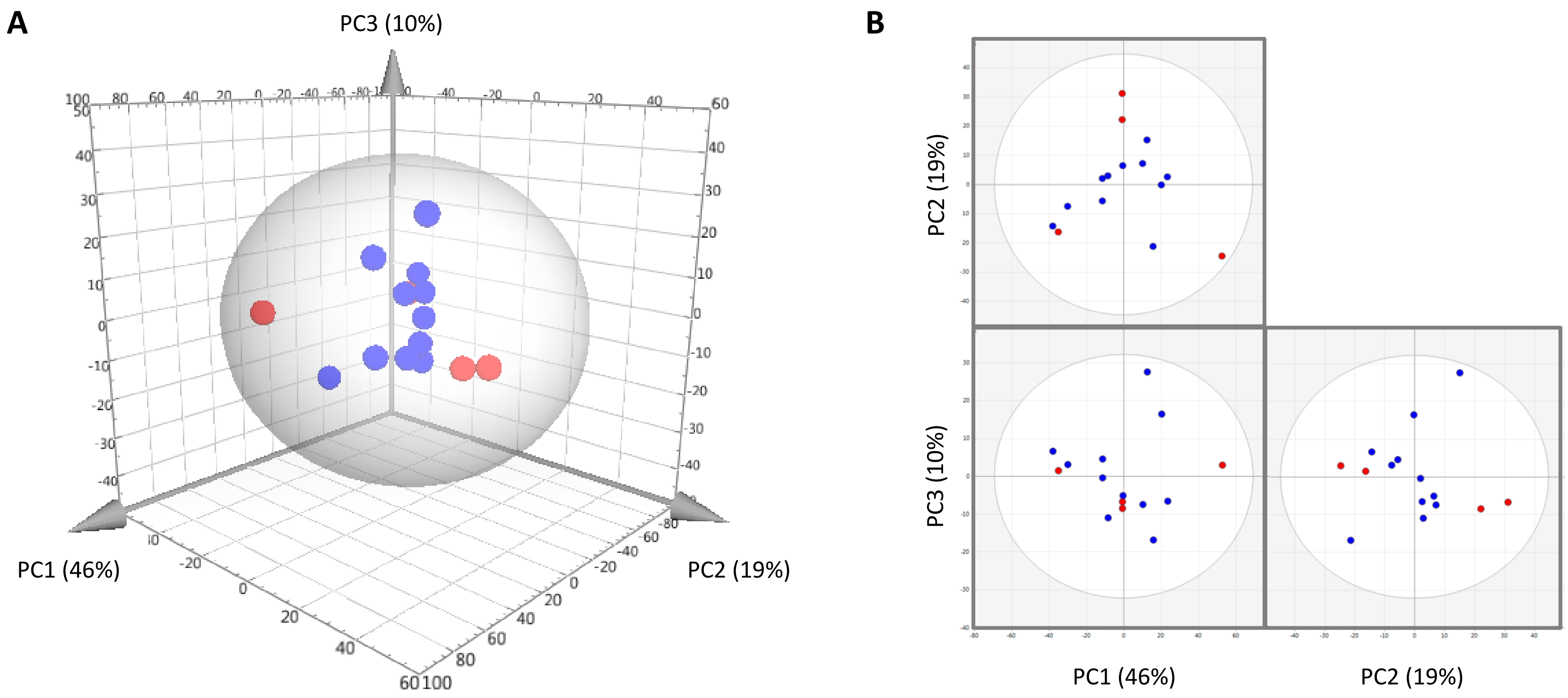

Supplementary Figure S1. Score plots of principal component analysis on gender distribution in high PISA group. Three-dimensional (A) and 2-dimensional (B) PC plots are shown. A total of 11 and 4 male and female subjects were included in high PISA group. The values for males and females are colored blue and red, respectively.

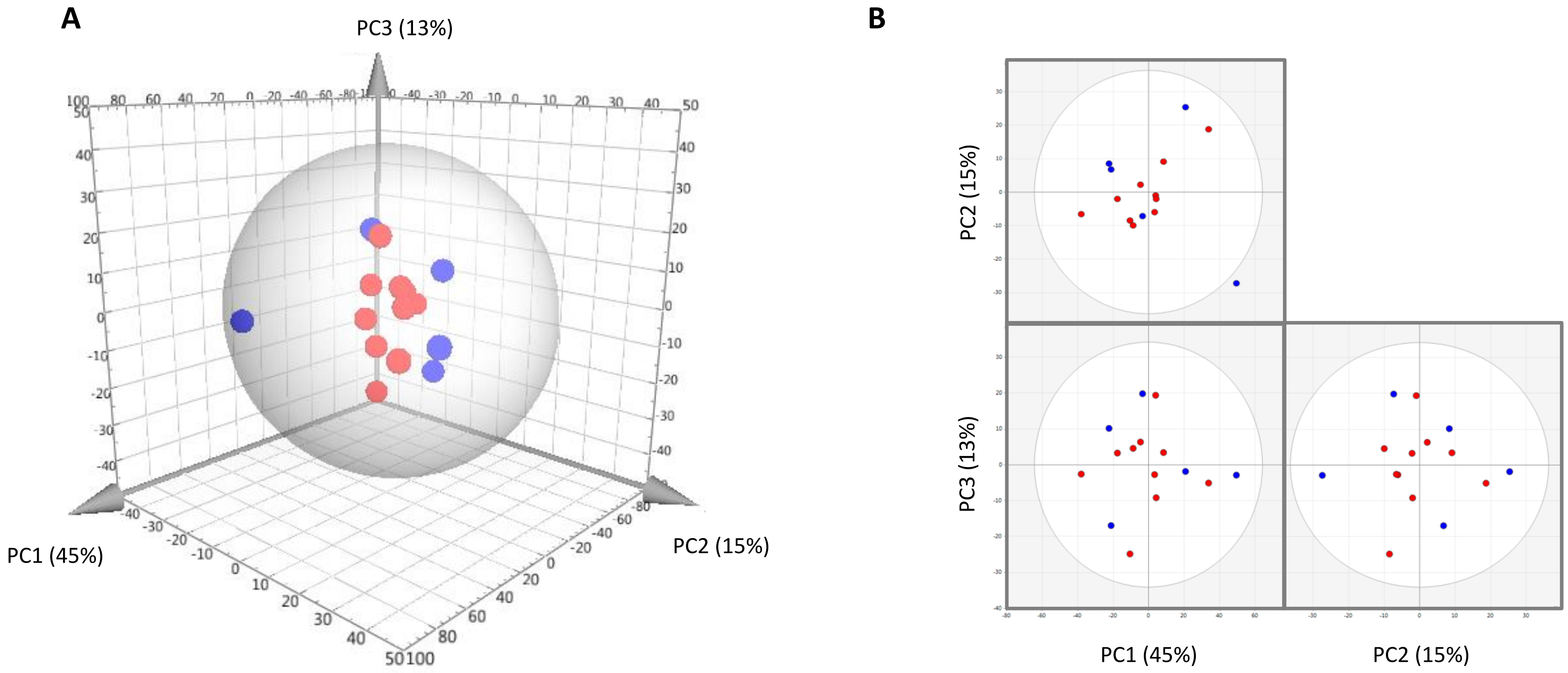

Supplementary Figure S2. Score plots of principal component analysis on gender distribution in low PISA group. Three-dimensional (A) and 2-dimensional (B) PC plots are shown. A total of 5 and 10 male and female subjects were included in low PISA group. The values for males and females are colored blue and red, respectively.

Supplementary Table S3. Comparison of the average peak intensity values of pre-debridement salivary metabolites according to gender

Metabolite	M/F[a]	*p* value[b]	
Propylene glycol	1.18	0.133	
Oxalacetic acid + Pyruvate	1.21	0.900	
Alanine_2TMS	1.35	0.318	
2-Hydroxybutyrate	1.31	0.109	
Sarcosine	0.764	0.148	
Leucine_1TMS	1.14	0.554	
2-Aminobutyric acid	1.23	0.443	
Isoleucine_1TMS	1.14	0.421	
Valine	1.20	0.421	
Serine_2TMS	1.40	0.318	
Ethanolamine	0.932	0.808	
Leucine_2TMS	1.28	0.554	
Isoleucine_2TMS	1.29	0.648	
Proline	1.29	0.503	
Succinic acid	1.28	0.190	
Glycine	1.18	0.455	
Glyceric acid	1.84	0.076	
Serine_3TMS	1.57	0.432	
Alanine_3TMS	1.37	0.165	
Threonine	1.40	0.607	
Thymine	1.22	0.421	
Hydrocinnamate	1.17	0.467	
b-Alanine	1.35	0.017	*

Aspartic acid	1.40	0.318
5-Oxoproline	1.16	0.432
4-Aminobutyric acid	0.993	0.778
Creatinine	0.877	0.047 *
3-Phenyllactic acid	1.06	0.421
Hypotaurine	1.04	0.648
Glutamic acid	1.41	0.218
5-Aminovaleric acid	0.983	0.778
Phenylalanine	1.12	0.691
4-Hydroxyphenylacetic acid	1.06	0.607
Lauric acid	0.983	0.808
Ribose	1.81	0.059
Fucose_1	1.04	0.677
Putrescine	0.939	0.764
Fucose_2	1.07	0.778
Glutamine	1.10	0.992
O-Phosphoethanolamine	1.05	0.479
Shikimic acid	1.32	0.299
Hypoxanthine	1.24	0.621
Ornithine	1.11	0.677
Isocitric acid + Citric acid	1.09	0.854
Cadaverine	1.53	0.105
Caffeine	1.13	0.118
1,5-Anhydro glucitol	1.07	0.691
Adenine	1.08	0.869

Fructose	12.1	0.734
Galactose	1.10	0.808
Glucose	1.44	0.101
Lysine	1.36	0.143
Histidine	1.28	0.109
Tyrosine	1.04	0.662
Paraxanthine	0.951	0.869
Indole-3-acetic acid	1.23	0.778
N-Acetylornithine	1.50	0.479
Xanthine	1.27	0.594
N-Acetylgalactosamine	1.33	0.594
Uric acid	1.09	0.720
Inositol	1.04	0.915
Arabinose-5-phosphate	1.60	0.101
Tryptophan	1.56	0.290
Spermidine	1.10	0.823
Fructose 6-phosphate	1.46	0.184
N-Acetylneuraminate	0.854	0.884
b-Lactose	0.916	0.749
Maltose	1.40	0.204
Lactitol	1.38	0.240

[a]M/F: the ratio of the average peak intensity values between samples from male and female subjects

[b]The *p* values were calculated by the Mann-Whitney test. $*p < 0.05$

Supplementary Table S4. Comparison of the average fold changes of salivary metabolites (pre-/post-debridement) according to gender

Metabolite	M/F[a]	*p* value[b]
Propylene glycol	0.978	0.900
Oxalacetic acid + Pyruvate	1.24	0.233
Alanine_2TMS	1.04	0.467
2-Hydroxybutyrate	0.656	0.290
Sarcosine	0.807	0.580
Leucine_1TMS	1.08	0.528
2-Aminobutyric acid	1.10	0.290
Isoleucine_1TMS	0.903	0.541
Valine	1.05	0.648
Serine_2TMS	1.11	0.635
Ethanolamine	0.954	0.705
Leucine_2TMS	0.998	0.528
Isoleucine_2TMS	0.729	0.869
Proline	0.888	0.705
Succinic acid	1.04	0.946
Glycine	0.953	0.930
Glyceric acid	1.27	0.035
Serine_3TMS	1.08	0.977
Alanine_3TMS	1.38	0.290
Threonine	0.983	0.854
Thymine	1.20	0.337
Hydrocinnamate	0.963	0.808
b-Alanine	1.06	0.479

Aspartic acid	0.982	0.946
5-Oxoproline	0.961	0.691
4-Aminobutyric acid	1.11	0.662
Creatinine	0.86	0.327
3-Phenyllactic acid	1.11	0.282
Hypotaurine	0.949	0.884
Glutamic acid	0.938	0.677
5-Aminovaleric acid	2.01	0.554
Phenylalanine	1.08	0.528
4-Hydroxyphenylacetic acid	2.03	0.749
Lauric acid	1.13	0.290
Ribose	1.18	0.621
Fucose_1	0.958	0.992
Putrescine	2.16	0.839
Fucose_2	1.01	0.528
Glutamine	0.67	0.357
O-Phosphoethanolamine	1.17	0.734
Shikimic acid	0.88	0.594
Hypoxanthine	1.19	0.884
Ornithine	0.995	0.900
Isocitric acid + Citric acid	1.09	0.479
Cadaverine	1.08	0.946
Caffeine	0.994	0.930
1,5-Anhydro glucitol	1.09	0.900
Adenine	1.18	0.410

Fructose	6.74	0.443
Galactose	1.14	0.607
Glucose	0.912	0.884
Lysine	0.853	0.869
Histidine	0.998	0.808
Tyrosine	0.986	0.823
Paraxanthine	1.03	0.854
Indole-3-acetic acid	1.10	0.516
N-Acetylornithine	0.828	0.491
Xanthine	1.06	0.823
N-Acetylgalactosamine	1.10	0.808
Uric acid	1.04	0.915
Inositol	1.07	0.491
Arabinose-5-phosphate	0.937	0.594
Tryptophan	1.06	0.503
Spermidine	0.968	0.961
Fructose 6-phosphate	0.999	0.607
N-Acetylneuraminate	1.07	0.607
b-Lactose	0.953	0.455
Maltose	1.02	0.677
Lactitol	0.963	0.793

[a]M/F: the ratio of the average fold changes between samples from male and female subjects

[b]The *p* values were calculated by the Mann-Whitney test.
